# Reconstructing a SuperGeneTree minimizing reconciliation

**DOI:** 10.1186/1471-2105-16-S14-S4

**Published:** 2015-10-02

**Authors:** Manuel Lafond, Aïda Ouangraoua, Nadia El-Mabrouk

**Affiliations:** 1 Département d'informatique et de recherche opérationnelle, Université de Montréal, Québec, Canada; 2 Département d'informatique, Université de Sherbrooke, Québec, Canada

**Keywords:** Super gene tree, Reconciliation, Supertree

## Abstract

Combining a set of trees on partial datasets into a single tree is a classical method for inferring large phylogenetic trees. Ideally, the combined tree should display each input partial tree, which is only possible if input trees do not contain contradictory phylogenetic information. The simplest version of the supertree problem is thus to state whether a set of trees is compatible, and if so, construct a tree displaying them all. Classically, supertree methods have been applied to the reconstruction of species trees. Here we rather consider reconstructing a super gene tree in light of a known species tree *S*. We define the supergenetree problem as finding, among all supertrees displaying a set of input gene trees, one supertree minimizing a reconciliation distance with S. We first show how classical exact methods to the supertree problem can be extended to the supergenetree problem. As all these methods are highly exponential, we also exhibit a natural greedy heuristic for the duplication cost, based on minimizing the set of duplications preceding the first speciation event. We then show that both the supergenetree problem and its restriction to minimizing duplications preceding the first speciation are NP-hard to approximate within a *n*^1-ϵ ^factor, for any 0 < ϵ < 1. Finally, we show that a restriction of this problem to uniquely labeled speciation gene trees, which is relevant to many biological applications, is also NP-hard. Therefore, we introduce new avenues in the field of supertrees, and set the theoretical basis for the exploration of various algorithmic aspects of the problems.

## Introduction

A fundamental task in evolutionary biology is to combine a collection of rooted trees on partial, possibly overlapping, sets of data, into a single rooted tree on the full set of data. This is the goal of supertree methods, mainly designed and used for the purpose of reconstructing a species supertree from a set of species trees (see overviews of early methods in [4-6], and more recent methods in [2,10,21,24,25,28,29]).

Ideally, the combined supertree should "display" each of the input tree, in the sense that by restricting the supertree to the leaf set of an input tree, we obtain the same input tree. However, this is not always possible, as the input trees may contain conflicting phylogenetic information. Note that considering a set of input trees that are not all compatible leads to the questions of correcting input gene trees or finding a subset of compatible input trees or subtrees [26]. Here, we leave open these questions and study the more direct formulation of the supertree problem that is to consider a set of compatible input trees and find a supertree displaying them all. The BUILD algorithm by Aho *et al*. [1] can be used to test, in polynomial time, whether a collection of rooted trees is compatible, and if so, construct a compatible supertree, not necessarily fully resolved. This algorithm has been generalized in [9,20] to output all compatible supertrees, and adapted in [27] to output all minimally resolved compatible supertrees.

Although supertree methods are classically applied to the construction of species trees, they can be used as well for the purpose of constructing gene trees. Several gene tree databases are available (see for example Ensembl Compara [30], Hogenom [22], Phog [11], MetaPHOrs [23], PhylomeDB [14], Panther[19]). For a gene family of interest, many different gene trees can therefore be available, and finding one single supertree displaying them all leads to a supertree question. On the other hand, given a gene of interest, a homology-based search tool is usually used to output all homologs in a set of genomes. The resulting gene family may be very large, involving distant gene sequences that may be hard to align, leading to weakly supported trees - or even worse, highly supported gene trees that are in fact incorrect. A standard way of reducing such errors is then to use a clustering algorithm based on sequence similarity, such as OrthoMCL [18], InParanoid [3], Proteinortho [17] or many others (see Quest for Orthologs links at http://questfororthologs.org/), to group genes into smaller sets of orthologs or inparalogs (paralogs that arose after a given speciation). Trees obtained for such partial gene families can then be combined by using a supertree method.

Considering input trees as parts of gene trees rather than as parts of species trees does not make any difference regarding the compatibility test procedure. However, for reconstructing a compatible "super gene tree", if a species tree is known for the taxa of interest, then it can be used as an additional information to choose among all possible supertrees displaying the input partial gene trees. Indeed, a natural optimization criterion is to minimize the reconciliation cost, i.e. either the duplication or the duplication plus loss cost, induced by the output tree. We call the problem of finding a compatible supertree minimizing a reconciliation cost *the supergenetree problem*.

In this paper, we first show how the exact methods developed for the supertree problem can be adapted to the supergenetree problem. As for the original algorithms, all the extensions have also exponential worst-time complexity. We then exhibit a heuristic, which can be seen as a greedy approach classically used for the supertree problems, that consists in constructing progressively the tree from its root to its leaves. The main module of this heuristic is to infer the minimum number of duplications preceding the first speciation, which we call the *Minimum pre-Speciation Duplication *problem. We show that the supergenetree problem for the duplication cost, and even its restricted version the Minimum pre-Speciation Duplication problem, are NP-hard to approximate within a *n*^1-ϵ ^factor, for any 0 < ϵ < 1 (*n *being the number of genes). Moreover, these inapproximability results even hold for instances in which there is only one gene per species in the input trees. Finally we consider the supergenetree problem with restrictions on input trees that are relevant to many biological applications. Namely, we require each gene to appear in at most one tree, and genes of any tree to be related through orthology only. This is for example the case of gene trees obtained for OrthoMCL clusters called orthogroups [18]. We show that even for this restriction, the supergenetree problem remains NP-hard for the duplication cost.

The following section introduces preliminary notations that will be required in the rest of the paper.

## Preliminaries

### Notations on trees

Given a set *L*, a *tree T for L *is a rooted tree whose leafset $L\left(T\right)$ is in bijection with *L*. We denote by *V*(*T*) the set of nodes and by *r*(*T*) the root of *T*. Given an internal node *x *of *T*, the subtree of *T *rooted at *x *is denoted *T_x_*. The *degree *of an internal node *x *of *T *is the number of children of *x*. If *T *is binary, we arbitrarily set one of the two children of *x *as the left child *x_l _*and the other as the right child *x_r_*. We call $\left(L\left(T_{x_{l}}\right),\;L\left(T_{x_{r}}\right)\right)$ the bipartition of a node *x *of degree 2 (note that the term 'bipartition' is sometimes used, in the context of unrooted trees, to denote the nodes or leaves of the two components obtained after removing a given edge. To avoid confusion, note that this is not what we mean here by 'bipartition').

A node *x *is an *ancestor *of *y *if *x *is on the (inclusive) path between *y *and the root, and we then call *y *a descendant of *x*. Two nodes *x *and *y *are *separated *in *T *if none is an ancestor of the other. The *lowest common ancestor *(lca) of a subset *L' *of $L\left(T\right)$, denoted *lca_T_*(*L'*), is the ancestor common to all nodes in *L' *that is the most distant from the root. The restriction *T*|_*L' *_of *T *to *L' *is the tree with leafset *L' *obtained from the subtree of *T *rooted at *lca_T_*(*L'*) by removing all leaves that are not in *L'*, and contracting all internal nodes of degree 2, except the root. We generalize this notation to a set of trees: For a set T  of trees on *L*, $T{|}_{L\prime }=\left\{T{|}_{L\prime }:T\in T\right\}$. Let *T' *be a tree such that L(T′)=L′⊆L(T). We say that *T *displays *T' *iff *T*|_*L' *_is the same tree as *T'*.

A *triplet *is a binary tree on a set *L *with |*L*| = 3. For *L *= {*x*, *y*, *z*}, we denote by *xy*|*z *the unique triplet *t *on *L *with root *r*(*t*) for which *lca_t_*(*x*, *y*) ≠ *r*(*t*) holds.

A *polytomy *(or star tree) over a set *L *is a tree for *L *with a single internal node, which is of degree |*L*|.

A *resolution B*(*T*) of a non-binary tree *T *is a binary tree respecting all the ancestral relations given by *T*. More precisely, *B*(*T*) is a binary tree such that $L\left(B\left(T\right)\right)=L\left(T\right)$, and for any *u*, *v *∈ *V*(*T*), if *u *is an ancestor of *v *in *T*, then $lca_{B\left(T\right)}\left(L\left(T_{u}\right)\right)$ is an ancestor of $lca_{B\left(T\right)}\left(L\left(T_{v}\right)\right)$.

### Gene and species trees

**Figure 1:**
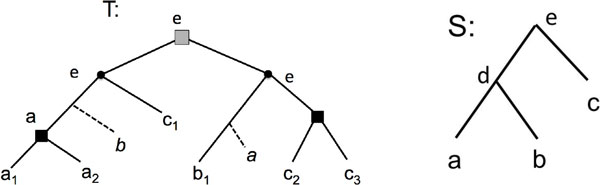
**A gene tree *T *for the gene family Γ = {*a*_1_, *a*_2_, *b*_1_, *c*_1_, *c*_2_, *c*_3_} and a species tree *S *for the set of species Σ = {*a*, *b*, *c*} and where, for any *x *and any *i*, *x_i _*is a gene in genome *x***. The label of an internal node *x *of *T *corresponds to *s*(*x*). Speciation nodes are represented by circles and duplication nodes by squares. The pre-speciation duplication nodes (here only one node) are grey-colored. The dotted lines represent losses that are inferred by a most parsimonious reconciliation algorithm. The duplication cost of *T *is 3 and its reconciliation cost is 5.

Figure 1 is an illustration of the notations defined in this section.

A species tree *S *for a set Σ = {*σ*_1_,⋯,*σ_t_*} of species represents an ordered set of speciation events that have led to Σ: an internal node is an ancestral species at the moment of a speciation event, and its children are the new descendant species. Inside the species' genomes, genes undergo speciation when the species to which they belong do, but also duplications and losses (other events such as transfers can happen, but we ignore them here). A *gene family *is a set of genes Γ accompanied with a *mapping function s *: Γ → Σ mapping each gene to its corresponding species.

Consider a gene family Γ where each gene *x *∈ Γ belongs to a species *s*(*x*) of Σ. The evolutionary history of Γ can be represented as a *gene tree T *for Γ, which is a rooted binary tree with its leafset in bijection with Γ, where each internal node refers to an ancestral gene at the moment of an event (either speciation or duplication). The mapping function *s *is generalized as follows: if *x *is an internal node of *T*, then $s\left(x\right)=lca_{S}\left(\left\{s\left(x'\right):x'\in L\left(T_{x}\right)\right\}\right)$.

An internal node *x *of *T *is called a *speciation node *if *s*(*x_l_*) and *s*(*x_r_*) are separated in *S*. Otherwise, *x *is a *duplication node *preceding the speciation event *lca_S_*(*s*(*x_l_*), *s*(*x_r_*)) if *lca_S_*(*s*(*x_l_*), *s*(*x_r_*)) is an internal node of *S*, otherwise it is a duplication inside the extant species *lca_S_*(*s*(*x_l_*), *s*(*x_r_*)). A duplication node *x *such that *s*(*x*) = *r*(*S*) is called a *pre-speciation duplication *node. A gene tree *T *with all internal nodes being speciation nodes is called a *speciation tree*. Two genes *x*, *y *of $L\left(T\right)$ are *orthologs in T *if their *lca_T_*(*x*, *y*) is a speciation node.

The *duplication cost *of *T *is the number of duplication nodes of *T*. It reflects the minimum number of duplications required to explain the evolution of the gene family inside the species tree *S *according to *T*. A well-known reconciliation approach [7,8] allows to further recover, in linear time, the minimum number of losses underlined by such an evolutionary history. We refer to the minimum number of duplications and losses required to explain *T *with respect to *S *as the *reconciliation cost *of *T *with respect to *S*, or simply the reconciliation cost if there is no ambiguity on the considered trees.

### Supergenetree problem statement

**Figure 2:**
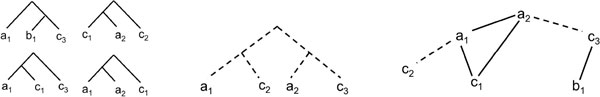
**Genes trees (left and middle) and their corresponding triplet graphs (right)**. Plain edges of the graph correspond to the four triplet trees, while dotted edges correspond to the triplets of the four-leaves tree.

A set G  of gene trees is said *consistent *if there is a tree *T*, called a *supergenetree *for G  displaying each tree of G , and *inconsistent *otherwise. A supergenetree *T *for G  is said *compatible *with G . For example, the four triplets in Figure 2 are consistent, and the gene tree *T *of Figure 1 is compatible with them. However, adding the dotted tree to the set of triplets makes the gene tree set incompatible. Consistency of a set of trees can be tested in polynomial time [1]. For a consistent set of trees, the problem considered here is to find a compatible gene tree of minimum reconciliation cost with respect to a given species tree. A formal statement of the general problem follows.

MINIMUM SUPERGENETREE PROBLEM (MINSGT PROBLEM):

**Input: **A species set Σ and a binary species tree *S *for Σ; a gene family Γ, a set Γ_*i*, 1 ≤ *i *≤ *k *_of subsets of Γ, and a set G  = {*G*_1_, *G*_2_,⋯, *G_k_*} of consistent gene trees where, for each 1 ≤ *i *≤ *k*, *G_i _*is a tree for Γ*_i_*.

**Output: **Among all gene trees for Γ compatible with G , one tree *T *of minimum reconciliation cost.

When the considered cost is the duplication cost, the problem is called the Minimum Duplication SuperGeneTree Problem (MinDUPSGT problem).

## From the SuperTree to the SuperGeneTree Problem

The classical supertree problem is to state whether or not a set of partial trees are consistent, and if so construct a tree containing them all. Here, we introduce the classical methods for solving this problem, and explore natural generalizations to the supergenetree problem.

Let Γ be a set of *n *taxa (usually species in case of the supertree problem, and genes in case of the supergenetree problem), Γ_*i*, 1 ≤ i ≤*k *_be a set of possibly overlapping subsets of Γ, and $G=\left\{G_{1},\;G_{2},\;\cdots \;,\;G_{k}\right\}$ be a set of trees where, for each 1 ≤ *i *≤ *k*, *G_i _*is a tree for Γ*_i_*. Let $tr\left(G\right)$ be the set of triplets of G  defined as $tr\left(G\right)=\left\{xy|z:\exists \;1\leq i\leq k\;\text{such}\;\text{that}\;G_{i}{|}_{\left\{x,\;y,\;z\right\}}=xy|z\right\}$. Let $T\left(\Gamma ,\;E\right)$ be the triplet graph with the set of vertices Γ and the set of edges *E *= {*xy *: ∃ *z *∈ Γ such that $xy|z\in tr\left(G\right)$} (see Figure 2 for an example).

**Figure 3:**
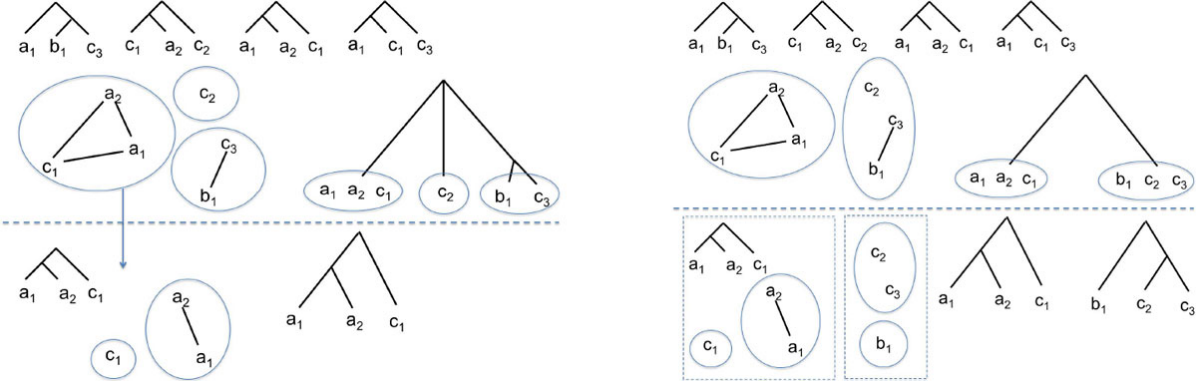
**Left: Execution of the BUILD algorithm on the set of given four triplets**. This example requires two iterations of the algorithm (delimited by a dotted line). At the first iteration, the triplet graph contains three components, leading to a polytomy with three leaves. The algorithm then iterates on the component {*a*_1_, *a*_2_, *c*_1_}, which terminates the supertree reconstruction procedure. Notice that the gene tree of Figure 1, which is compatible with the four triplets, is not a resolution of this non-binary tree; Right: A variant of the BUILD algorithm, with the triplet graph components grouped into bipartitions - in this case leading to a fully resolved tree. This tree is actually the gene tree *T *of Figure 1.

The classical BUILD algorithm [1] determines, in polynomial time, whether a set of triplets is consistent and if so constructs a tree *T*, possibly non-binary, compatible with them. The algorithm takes as input the graph $T=T\left(\Gamma ,\;E\right)$. Let $C\left(T\right)=\left\{C_{1},\;\cdots \;,\;C_{m}\right\}$ be the set of connected components of T . If T  has at least three vertices and $|C\left(T\right)|=1$, then G  is inconsistent, and the algorithm terminates. For example, the set of five gene trees of Figure 2 is inconsistent, as the corresponding triplet graph (including dotted lines) is connected. Otherwise, if $|V\left(T\right)|\geq 3$, a polytomy is created over $C\left(T\right)$, the internal node of the polytomy being the root *r*(*T*) of the compatible tree *T *under construction and its children being *m *subtrees with leafsets *V*(*C*_1_),..., *V*(*C_m_*), with their topology yet to be determined (where *V*(*C_i_*) ⊆ Γ denotes the set of taxa appearing in *C_i_*). The algorithm then recurses into each connected component, i.e. the subtree for *V*(*C_i_*) is determined recursively from the graph $T\left(V\left(C_{i}\right),\;E{|}_{C_{i}}\right)$ defined by *E*|*_C_i __*= {*xy *: ∃ *z *∈ Γ such that $xy|z\in tr\left(G{|}_{V\;\left(C_{i}\right)}\right)$}. If, at any step, the considered graph has a single component containing more than two vertices, then G  is reported as an inconsistent set of trees and the algorithm terminates. Otherwise, recursion terminates when the graph has at most two vertices, eventually returning a supertree *T*. See Figure 3 for an example.

The BUILD algorithm has been generalized in an algorithm called AllTrees [20] to output all supertrees compatible with a set of triplets in case consistency holds. Instead of taking each element of $C\left(T\right)$ as a separate leaf of *r*(*T*), all possible groupings, in other words all partitions of $C\left(T\right)$, are considered (see Figure 3, right, for a choice of bipartitions). For each partition $P\left(C\left(T\right)\right)$ of $C\left(T\right)$, a polytomy is created over $P\left(C\left(T\right)\right)$. The algorithm then iterates by considering each possible partition of each subgraph induced by each element of $P\left(C\left(T\right)\right)$. The algorithm is polynomial in the size of the output that may be exponential in the size of the input.

A tree *T *compatible with G  such that no internal edge of *T *can be contracted so that the resulting tree is also compatible with G  is called a minimally resolved supertree. Minimally resolved supertrees contain all the information about all supertrees compatible with G  but in a "compressed" format. By exhibiting some properties on graph components, Semple shows in [27] how some partitions of the triplet graph components can be avoided without loss of generality. The new developed algorithm, named AllMinTrees [27], outputs a minimally resolved tree in polynomial time. However, it was shown in [15] that the cardinality of the solution space can be exponential in *n *= |Γ|, leading to an exponential time algorithm with $\Omega \left({\frac{n}{2}}^{\frac{n}{2}}\right)$.

Notice that, in general, the trees output by all these methods are non-binary trees.

### Extensions to the SuperGeneTree problem

Natural exact solutions for the supertree problem can be extended to the supergenetree problem as follows:

(1) Use AllMinTrees to output all minimally resolved supertrees, and for each one which is non-binary in general, find in linear time a resolution minimizing the reconciliation [16,32] or duplication [31] cost. Among all optimally resolved trees, select one of minimum cost. Clearly this approach has the same complexity as the AllMinTrees algorithm, multiplied by a factor of *n *to resolve each tree, which is $\Omega \left(n\cdot {\frac{n}{2}}^{\frac{n}{2}}\right)$.

(2) As we are seeking a binary tree, each created node *x *of the supergenetree *T *under construction should determine a bipartition $\left(L\left(T_{x_{l}}\right),\;L\left(T_{x_{r}}\right)\right)$. Therefore, the AllTrees algorithm can be simplified by considering, instead of all partitions of $C\left(T\right)$, only all bipartitions of the triplet graph components set. See an example in Figure 3, right. Notice that this simplification approach is not applicable to the AllMinTrees algorithm, as by imposing bipartitions, the minimum resolution condition cannot be guaranteed.

### A branch-and-bound approach

The tree space which is explored by the two exact methods described above can be reduced by using a branch-and-bound approach. Consider for example method (1) using the AllMinTrees algorithm. At each iteration of computing one minimally resolved tree, resolve the intermediate non-binary tree obtained at this step, using for example the linear-time algorithm presented in [16]. If its reconciliation cost is greater than the cost of a full tree already obtained at a previous stage of the AllMinTrees algorithm, then stop expanding this tree as this can only increase the reconciliation cost.

### A dynamic programming approach

The recursive top-down method (2) can instead be handled by a dynamic programming approach computing the minimum reconciliation cost of a tree on a subset of Γ according to the reconciliation costs of trees on smaller subsets, similarly to the wrok done in [13].

More precisely, let *P *be an arbitrary subset of Γ, and denote by *R*(*P*) the minimum duplication cost of a tree *T*|*_P _*having leafset *P *and compatible with the set $G{|}_{P}=\left\{G_{1}{|}_{P},\;G_{2}{|}_{P},\;\cdots \;,\;G_{k}{|}_{P}\right\}$. Let $T\left(P,\;E{|}_{P}\right)$ be the BUILD graph restricted to *P *and $G{|}_{P}$, and $C\left(T\right)=\left\{C_{1},\;\cdots \;,\;C_{m}\right\}$ the set of its connected components. If $C\subseteq C\left(T\right)$, by *V*(*C*) we mean $\cup_{C_{i}\in C}V(C_{i})$. Denote the complement of *C *by $\bar{C}=C\left(T\right)\backslash C$. Finally set $d(V(C),\;V(\bar{C}))$ to 0 if *s*(*V*(*C*)) and $s(V(\bar{C})$) are separated in *S*, in which case (*V*(*C*), $V(\bar{C})$ is the bipartition of a speciation node, and 1 otherwise i.e. if (*V*(*C*), $V(\bar{C})$ is the bipartition of a duplication node. Then:

$$R\left(P\right)=\underset{C\subset C\left(T\right)}{\text{min}}R\left(V\left(C\right)\right)+R\left(V\left(\bar{C}\right)\right)+d\left(V\left(C\right),\;V\left(\bar{C}\right)\right)$$

the value of interest being *R*(Γ). First note that, assuming constant-time *lca *queries over *S*, *d*(*V*(*C*), $V(\bar{C})$ can be computed in constant time if *s*(*V*(*C*)) and $s(V(\bar{C}))$ can be accessed in constant time, since if suffices to check that the *lca *of *s*(*V*(*C*)) and $s(V(\bar{C}))$ differs from both. To achieve this, we precompute *s*(*X*) for every subset *X *of Γ of size 1, 2,..., *n *in increasing order. Noting that if |*X*| > 1, then for any *x *∈ *X*, *s*(*X*) = *lca_S_*(*s*(*X *\ {*x*}), *s*(*x*)), *s*(*X*) can be computed in constant time assuming that *s*(*X *\ {*x*}) was computed previously and assuming constant-time *lca *queries. As there are 2*^n ^*subsets of Γ, each computed in constant time, this preprocessing step takes time *O*(2*^n^*).

As for *R*(Γ), we can simply ensure that each *R*(*P*) is computed at most once by storing its value in a table for subsequent accesses (i.e. when *R*(*P*) is needed, we use its value if it has been computed, or compute it and store it otherwise). In this manner, each subset *P *takes time, not counting the recursive calls, proportional to $|P||G|+|P|+2^{\left|C\left(T\right)\right|}$ to construct $T\left(P,\;E{|}_{P}\right)$, find $C\left(T\right)$, and evaluate each bipartition of $C\left(T\right)$. We will simply use the fact that $|P||G|+|P|+2^{\left|C\left(T\right)\right|}$ is in *O*(2*^n^*). As this has to be done for, at worst, each of the 2*^n ^*subsets of Γ, we get a total time *O*(2*^n ^*+ 2*^n ^*·2*^n^*) = *O*(4*^n^*). Note that this analysis probably overestimates the actual complexity of the algorithm, as we are assuming that each subset *P *and each component set $C\left(T\right)$ are both always of size *n*. It is also worth mentioning that the *R*(*P*) recurrence can easily adapted to the mutation cost (duplications + losses).

### A greedy heuristic for the duplication cost

Instead of trying all partitions of the triplet graph components set at each step of the AllTrees or AllMinTrees algorithms, if the goal is to minimize the duplication cost, then a natural greedy approach would be to choose the best partition at each iteration, namely the one allowing to minimize the number of duplications preceding each speciation event. Such an approach would result in pushing duplications down the tree. It leads to the following restricted version of the supergenetree problem.

MINIMUM PRE-SPECIATION DUPLICATION PROBLEM (MINPRESPEDUP PROBLEM):

**Input: **A species set Σ and a binary species tree *S *for Σ; a gene family Γ, a set Γ_*i*,1≤*i*≤*k *_of subsets of Γ, and a set $G=\left\{G_{1},\;G_{2},\;\cdots \;,\;G_{k}\right\}$ of consistent gene trees where, for each 1 ≤ *i *≤ *k*, *G_i _*is a tree on Γ*_i_*.

**Output: **Among all gene trees for Γ compatible with G , one tree *T *with minimum pre-speciation duplication nodes.

We will show in the following section that even this restricted version of the supergenetree problem is hard. Here, we give the intuition of a natural way of solving this problem, that reduces to repeated applications of the Max-Cut problem. Although known to be NP-hard, efficient heuristics exist (up to a factor of 0.878 [12]), that can be used for our purpose.

**Figure 4:**
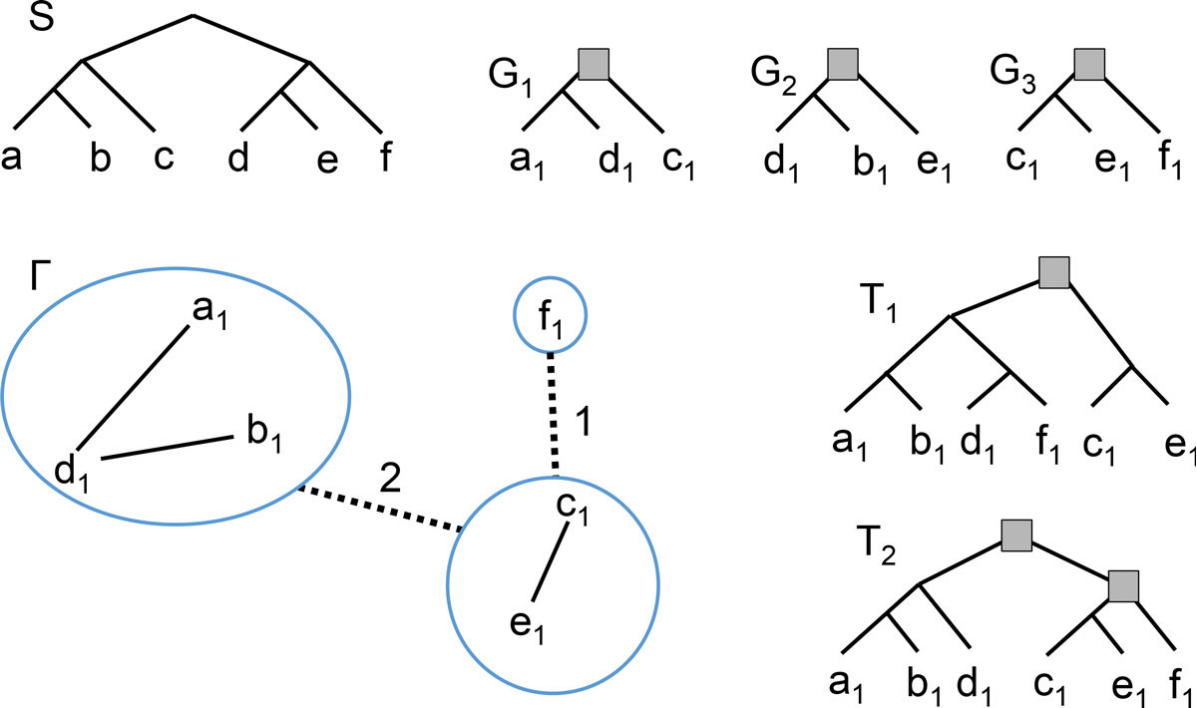
**Example of how Max-Cut can be applied to the MinPreSpeDup problem**. *S *is a species tree, $G=\left\{G_{1},\;G_{2},\;G_{3}\right\}$ and T  is the BUILD graph (solid edges). Its connected components are enclosed in circles, and the dotted edges represent required duplications mapped to *r*(*S*). The edge of weight 2 is explained by the *a*_1_*d*_1_|*c*_1 _and *d*_1_*b*_1_|*e*_1 _triplets, whereas the edge of weight 1 is explained by the *c*_1_*e*_1_|*f*_1 _triplet. A Max-Cut creates the bipartition ({*a*_1_, *b*_1_, *d*_1_, *f*_1_}, {*c*_1_, *e*_1_}), leading to the *T*_1 _tree which merges all required duplications at its root. The tree *T*_2 _is obtained from the suboptimal bipartition ({*a*_1_, *b*_1_, *d*_1_}, {*c*_1_, *e*_1_, *f*_1_}) and has 2 duplications.

For the supertree problem, the triplet graph $T=T\left(\Gamma ,\;E\right)$ represents all triplets of the input trees that have to be combined. In the case of the supergenetree problem, another tree is available, the species tree *S*. A triplet *xy*|*z *found in the input trees G  can be reconciled with *S*, and if *r*(*xy*|*z*) is a duplication, then any tree compatible with *G *must contain this duplication. Say that *r*(*xy*|*z*) is a required duplication mapped to *r*(*S*) if *s*(*r*(*xy*|*z*)) = *r*(*S*) and *r*(*xy*|*z*) is a duplication. Let us include this information in T . More precisely, let $C=C\left(T\right)$ denote the set of connected components of T , and let $T\left(C\right)$ be the graph whose vertex set is C , and $C_{1},\;C_{2}\in C$ share an edge if *C*_1 _has vertices *x*, *y *and *C*_2 _has a vertex *z *such that *xy*|*z *is a triplet in G  with *r*(*xy*|*z*) being a required duplication mapped to *r*(*S*). If there are, say, *d *distinct such triplets, one can possibly set a weight of *d *to the *C*_1_*C*_2 _edge. See Figure 4 for an example.

Consider the problem of clustering the components of $T\left(C\right)$ into two parts *B*_1_, *B*_2 _of a bipartition in a way minimizing the number of duplications preceding the speciation event *r*(*S*). For each *C*_1 _∈ *B*_1 _and *C*_2 _∈ *B*_2 _such that *C*_1_*C*_2 _is an edge of $T\left(C\right)$, a tree *T *rooted at the bipartition (*B*_1_, *B*_2_) contains the required duplications mapped to *r*(*S*) represented by the *C*_1_*C*_2 _edge. If there are *k *such edges between *B*_1 _and *B*_2 _totalizing a weight of *w*, the single duplication at the root of *T *contains those *w *required duplications. In other words, we have "merged" *w *required duplications into one. It then becomes natural to find the bipartition of $T\left(C\right)$ that merges a maximum of duplications, i.e. that contains a set of edges crossing between the two parts of maximum weight. This is the well-known Max-Cut problem. For instance in Figure 4, the Max-Cut has a weight of 3 and leads to the optimal tree *T*_1_. Any other bipartition sends a required duplication to a lower level and is hence suboptimal. The *T*_2 _tree is obtained from first taking the suboptimal ({*a*_1_, *b*_1_, *d*_1_}, {*c*_1_, *e*_1_, *f*_1_}) bipartition, which creates a duplication at the root and defers the *c*_1_*e*_1_|*f*_1 _required duplication for later.

Note however that the components of T  may contain required duplications themselves, which are not represented by the edges of $T\left(C\right)$. Thus, a Max-Cut must then be applied recursively on both parts of the chosen bipartition. Therefore, this method does not benefit directly from the efficient approximation factor known for the Max-Cut problem, as the approximation error stacks with each application. In the next section, we show that, unlike Max-Cut, the MinPreSpeDup problem cannot admit a constant factor approximation (unless P = NP).

## Inapproximability of the MinDupSGT and MinPreSpeDupSGT problems

Through the rest of this section, we denote by *n *= |Γ| the size of the considered gene family. We show that both the MinDupSGT problem and its restriction the MinPreSpeDupSGT problem are NP-hard.

**Theorem 1 ***The *MinDupSGT *and *MinPreSpeDupSGT *problems are both NP-hard to approximate within a factor of n*^1-ϵ ^*for any constant *0 < ϵ < 1. *Moreover*, *this result holds for both problems even when restricted to instances having at most one gene per species in *Γ.

*Proof *We use a reduction from the minimum *k*-colorability problem. Recall that a graph *H *= (*V*, *E*) is *k*-colorable if there is a partition {*V*_1_, *V*_2_,..., *V_k _*} of *V *into independent sets (i.e. if *x*, *y *∈ *V_i _*for some 1 ≤ *i *≤ *k*, then *xy *∉ *E*). It is now well-known [33] that the smallest *k *for which *H *is *k*-colorable cannot be approximated within a factor of |*V*|^1-ϵ ^unless *P *= *NP*.

Now, given a graph *H *= (*V*, *E*), we construct a gene set Γ, a set of rooted triplet gene trees G  and a species tree *S *such that *H *is *k*-colorable if and only if G  is compatible with some gene tree *T *having at most *k − *1 duplications when reconciled with *S*. Using the same construction, we also show that *H *is *k*-colorable if and only if G  is compatible with some gene tree *T *having at most *k *− 1 pre-speciation duplications when reconciled with *S*. In both cases, the gene-species mapping *s *is bijective, proving the second part of the theorem statement.

Let Γ = {*v*_1_, *v*_2 _: *v *∈ *V*} and for each edge *vw *∈ *E*, add the triplets *v*_1_*v*_2_|*w*_1_, *v*_1_*v*_2_|*w*_2_, *w*_1_*w*_2_|*v*_1 _and *w*_1_*w*_2_|*v*_2 _to G . Observe that this forces any tree *T *that displays G  to display the tree ((*v*_1_, *v*_2_), (*w*_1_, *w*_2_)). Add one species to Σ for each gene of Γ so that the gene-species mapping *s *is bijective. As for *S*, first let *S*_1 _be any binary tree with one leaf for each member of {*s*(*v*_1_) : *v *∈ *V*}, and in the same manner let *S*_2 _be any binary tree with one leaf for each member of {*s*(*v*_2_) : *v *∈ *V*}. Obtain *S *by connecting the root of *S*_1 _and the root of *S*_2 _under a common parent *r*(*S*). Thus *s*(*v*_1_) and *s*(*v*_2_) are separated by *r*(*S*) for any *v *∈ *V*. Clearly, G  and *S *can be constructed in polynomial time.

**Claim 1 **: if *H *is *k*-colorable, then we can find a tree *T *compatible with G  having at most *k − *1 duplications. Moreover each such duplication *x *is a pre-speciation duplication (i.e. *s*(*x*) = *r*(*S*)).

Let {*V*_1_, *V*_2_,..., *V*_*k*_} be a *k*-coloring of *H*. For each 1 ≤ *i *≤ *k*, let *T_i _*be the tree with leafset $V_{i}^{\prime }=\left\{v_{1},\;v_{2}:v\in V_{i}\right\}$ that has only speciations, i.e. *T_i _*is $S{|}_{s\left(V_{i}^{\prime }\right)}$ (because all genes in *V'*_*i *_belong to a different species). Notice that *s*(*r*(*T_i_*)) = *r*(*S*), since *r*(*S*) separates *v*_1 _from *v*_2 _for all *v *∈ *V*. Obtain *T *by taking any binary tree on *k *leaves (and hence *k *− 1 internal nodes), then replacing each leaf by a distinct *T_i_*. In this manner, *T *has *k *− 1 duplications since only the internal nodes of *T *that do not belong to any *T_i _*need to be duplications. Moreover, each duplication node *x *has *s*(*x*) = *r*(*S*). It remains to show that *T *is compatible with G . It suffices to observe that all triplets of G  are of the form *v*_1_*v*_2_|*w_h _*with *h *∈ {1, 2}, and that such a triplet being in G  implies that *vw *∈ *E*. For such a triplet, we must then have *v *∈ *V_i _*and *w *∈ *V_j _*with *i *≠ *j*, implying $v_{1},\;v_{2}\in V_{i}^{\prime }$ and $w_{h}\in V_{j}^{\prime }$. By the construction of *T*, *v*_1_*v*_2_|*w_h _*must be a triplet of *T*, as desired.

**Claim 2 **: if there is a tree *T *compatible with G  having *k *− 1 duplications, then *H *is *k*-colorable. Moreover if *T *has *k *−1 duplications such that each duplication *x *has *s*(*x*) = *r*(*S*), then *H *is *k*-colorable.

Let *T *be a tree compatible with G  having *k *− 1 duplications. Call a node *x *of *T S-maximal *if *x *is not a duplication node mapped to *r*(*S*) but every proper ancestor of *x *is a duplication mapped to *r*(*S*). Let *X *= {*x*_1_, *x*_2_,..., *x_m_*} be the set of *S*-maximal nodes of *T*. Note that if *y *≠ *r*(*T*) is a duplication mapped to *r*(*S*), then so is the parent of *y*. This implies that every leaf ℓ of *T *has at least one ancestor *x_i _*in *X*, since *x_i _*is the highest (i.e. closest to the root) ancestor of ℓ that is not a duplication mapped to *r*(*S*) (such an *x_i _*always exists, since ℓ is itself one such node). Moreover, *x_i _*is unique, as no other *x_j _*∈ *X *can be the ancestor of *x_i_*. Therefore, $\left\{L\left(T_{x_{1}}\right),\;\cdots \;,\;L\left(T_{x_{m}}\right)\right\}$ is a partition of $L\left(T\right)$. We next show that *m *≤ *k*. Let *T' *be the tree obtained by removing all descendants of *x_i _*in *T*, for all 1 ≤ *i *≤ *m*. Then *T' *is a binary tree with *m *leaves, and all its *m *− 1 internal nodes are duplications mapped to *r*(*S*). Since *T *has no more than *k *− 1 duplications (in either cases of the claim), *T' *has at most *k *− 1 internal nodes and therefore at most *k *leaves. We deduce that *m *≤ *k*.

Observe that if *vw *∈ *E*, then *α *= *lca*(*v*_1_, *v*_2_, *w*_1_, *w*_2_) must be a duplication such that *s*(*α*) = *r*(*S*). Indeed, *α *separates *lca*(*v*_1_, *v*_2_) from *lca*(*w*_1_, *w*_2_) since *T *displays ((*v*_1_, *v*_2_), (*w*_1_, *w*_2_)). But since *s*(*lca*(*v*_1_, *v*_2_)) = *s*(*lca*(*w*_1_, *w*_2_)) = *r*(*S*) by the construction of *S*, *s*(*α*) can only be *r*(*S*) as well, and so *α *must be a duplication.

Now, let *V_i _*= {*v *: *v*_1 _is a descendant of *x_i_*} for each 1 ≤ *i *≤ *m*. Take *v*, *w *∈ *V_i _*for some *i*. We show that *vw *∉ *E*, and thus that {*V*_1_,..., *V_m_*} forms a coloring of *H *with at most *k *colors. The argument applies whether each duplication maps to *r*(*S*) or not, proving both parts of the claim. Suppose for the sake of contradiction that *vw *∈ *E*, but *v*, *w *∈ *V_i_*. In *T*, *lca*(*v*_1_, *w*_1_) must be a descendant of *x_i_*, since *x_i _*is a common ancestor of *v*_1 _and *w*_1 _by the definition of *V_i_*. Moreover, *lca*(*v*_1_, *w*_1_) ≠ *x_i _*since *lca*(*v*_1_, *w*_1_) = *lca*(*v*_1_, *v*_2_, *w*_1_, *w*_2_) is a duplication mapped to *r*(*S*), as shown above, while *x_i _*is not such a duplication, by its definition. Therefore, *lca*(*v*_1_, *w*_1_) is a proper descendant of *x_i_*. But *s*(*lca*(*v*_1_, *w*_1_)) = *r*(*S*) = *s*(*x_i_*) implies that *x_i _*is a duplication mapped to *r*(*S*), a contradiction. We conclude that {*V*_1_,..., *V_m_*} with *m *≤ *k *is a proper coloring of *H*.

This reduction, together with the fact that the *k*-coloring problem is NP-hard to approximate within a *n*^1-ϵ ^factor, proves the Theorem.   □

## Independent Speciation trees

We now consider the MinDupSGT problem in the special case where the input gene trees are *independent speciation trees*, meaning: (1) each gene of Γ appears in at most one gene tree leafset, and (2) gene trees of $G=\left\{G_{1},\;G_{2},\;\cdots \;,\;G_{k}\right\}$ are all speciation trees with respect to the species tree *S*. Our objective is to find a gene tree *T *compatible with G  minimizing duplications that also maintains the orthology relationships specified by G . In other words, we require that for every $G_{i}\in G,\;T{|}_{L\left(G_{i}\right)}$ has only speciations. We say that a gene tree *T *that satisfies this property *preserves the speciations *of G . Note that if *T *preserves the speciations of G , then it is necessarily compatible with G . We call $T{|}_{L\left(G_{i}\right)}$ the *copy of G_i _*in *T*.

MINIMUM SPECIATION SUPERGENETREE (MINSPECSGT PROBLEM):

**Input: **A species set Σ and a binary species tree *S *for Σ; a gene family Γ, a set Γ_*i*,1≤*i*≤*k *_of disjoint subsets of Γ, and a set $G=\left\{G_{1},\;G_{2},\;\cdots \;,\;G_{k}\right\}$ of consistent *independent speciation trees *such that, for each 1 ≤ *i *≤ *k*, *G_i _*is a tree for Γ*_i_*.

**Output: **Among all gene trees for Γ that preserve the speciations of G , one tree *T *of minimum duplication cost.

Notice that, since no gene of Γ appears more than once in the set of input trees, G  always admits a solution. Indeed, taking any binary tree on *k *leaves and replacing each leaf by a distinct *G_i _*achieves the desired result. However, while apparently easier, we show that finding such a gene tree *T *minimizing the number of duplications is still hard.

**Theorem 2 ***The decision version of the MinSpecSGT problem is NP-Complete*, *i.e. it is NP-Complete to decide if a species tree S and a set of independent speciation trees G admit a supertree T that preserves its speciations with at most k duplications*.

*Proof *The problem is easily seen to be in NP, as it is easy to verify in polynomial time that a given gene tree *T *is compatible with G , preserves its speciations and has *k *duplications. For NP-hardness, we turn to the decision version of the *k*-colorability problem. That is, for a given *k*, deciding if a graph *H *= (*V*, *E*) is *k*-colorable is NP-hard. We create from *H *a species tree *S *and a set of independent speciation trees G  such that *H *is *k*-colorable if and only if *S *and *G *admit a supertree *T *with at most *k − *1 duplications.

Let *n *= |*V*|, and denote *V *= {*v*_1_,..., *v_n_*}. To create *S*, start with any binary tree *S' *on $\left(\begin{array}{c} n\\ 2 \end{array}\right)$ leaves. denote this leafset *W *= {*w_i,j_*: 1 ≤ *i *<*j *≤ *n*} so that there is a one-to-one correspondence between *W *and the unordered pairs of *V*. Then, add a special leaf *a *by joining it with the root of *S' *under a common parent *p*, and finally obtain *S *by adding another special leaf *b *by joining it with *p *under a common parent. Therefore, the species set is $\sum =L\left(S\right)=W\cup \left\{a,\;b\right\}$.

For the construction of each gene tree G∈G, we ease up notation by labeling each leaf *g *of *G *by *s*(*g*) directly (e.g. if we say that *G *is of the form (*a*, *b*), we mean that G  has two leaves *g_a_*, *g_b _*such that *s*(*g_a_*) = *a *and *s*(*g_b_*) = *b*). In this manner, since all trees of G  contain only speciations, each tree G∈G must be a subtree of *S *(or it is obtained from such a subtree by contracting edges). Also recall that we are assuming that each gene appears in at most one gene tree of G , and so the genes from two distinct trees must also be distinct (even if they share the same label).

In G , we first add *k *trees of the form (*a*, *b*), plus one tree *G_i _*for each vertex *v_i _*in *H*. The tree *G_i _*corresponding to *v_i _*∈ *V *is a copy of *S *from which we remove every leaf except those *w_j,k _*for which one of *j *= *i *or *k *= *i *holds, and *(v_j _v_k) _*∈ *E *(i.e. we keep the leaves of *W *that correspond to an edge incident to *v_i_*). Also contract the degree 2 nodes of *G_i_*. Notice that if *v_i_v_j _*∈ *E *and *i *<*j*, then both *G_i _*and *G_j _*contain a gene in the *w_i,j _*species. Also, if *v_i_v_j _*∉*E *then *G_i _*and *G_j _*have no genes from a common species.

**Claim 1 **: if *H *is *k*-colorable, then *S *and G  admit a supertree *T *having at most *k *− 1 duplications.

Let {*V*_1_,..., *V_k _*} be a *k*-partition of *V *into independent sets. Take any *h *such that 1 ≤ *h *≤ *k*. Recall that if *v_i_*, *v_j _*∈ *V_h_*, then *G_i _*and *G_j _*share no gene from a common species (since *v_i_v_j _*∉ *E*). Thus the trees in $G_{h}=\{G_{i}:v_{i}\in V_{h}\}$ are all disjoint in terms of species. Let Σ*_h _*be the set of species that appear in some tree of $G_{h}$. Then, the tree $S{|}_{\Sigma_{h}}$ contains a copy of each tree in $G_{h}$, and none of these copies overlap. Obtain *T_h _*by joining a gene labeled *a *to *r*($S{|}_{\Sigma_{h}}$) under a common parent *p*, then joining a gene labeled *b *to *p *under a new common parent. Now, *T_h _*contains a copy of each tree in *G_h _*and a copy of one of the (*a*, *b*) trees. By taking a tree with *k *leaves (where at worst, each *k *− 1 internal node is a duplication), and replacing each leaf by the speciation trees *T*_1_,..., *T_k_*, we obtain a gene supertree *T*, which preserves the speciations of G  and has at most *k *− 1 duplications.

**Claim 2 **: if *S *and G  admit a supertree *T *having *k − *1 duplications, then *H *is *k*-colorable.

We first show that if *T *has *k *− 1 duplications, then it must have exactly *k *speciations mapped to *r*(*S*). It cannot have more, as there would then be more than *k *− 1 duplications. Suppose instead that there are *k' *<*k *such speciations, and denote them *x*_1_,...,*x_k'_*. Note that there must be at least *k' *− 1 duplications in the ancestors of the *x_i_*s. Now, for 1 ≤ *i *≤ *k'*, $T_{x_{i}}$ must contain a certain number of copies of *a *and *b*. Let *m_i_*(*a*) and *m_i_*(*b*) denote, respectively, the number of copies of *a *and *b *contained in $T_{x_{i}}$, noting that in total, there are *k *copies of each since there are *k *subtrees of the form (*a*, *b*) in G . Since *x_i _*is a speciation mapped to *r*(*S*), it separates the *a *copies from the *b *copies, thus the $T_{x_{i}}$ subtree must contain at least *m_i_*(*a*) − 1 + *m_i_*(*b*) − 1 duplications. Denote by *d*(*T*) the number of duplications in *T*. It follows that $\begin{array}{c} d\left(T\right)\geq k^{\prime }-1+\sum_{i=1}^{k^{\prime }}\left(m_{i}\left(a\right)+m_{i}\left(b\right)-2\right)=k^{\prime }-1-2k^{\prime }+\sum_{i=1}^{k^{\prime }}m_{i}\left(a\right)+\sum_{i=1}^{k^{\prime }}(m_{i}\left(b\right)\\ =-k^{\prime }-1+k+k=2k-k\prime -1>k-1 \end{array}$ when *k' *<*k*, a contradiction.

Now, we can let *x*_1_,..., *x_k _*be the *k *speciation nodes of *T *mapped to *r*(*S*). The *k − *1 duplications of *T *must then all be ancestors of the *x_i_*, and they are all mapped to *r*(*S*). Therefore the $T_{x_{1}},...,T_{x_{h}}$ subtrees each contain only speciations. For any $G_{i}\in G$ corresponding to *v_i_*, one of the $T_{x_{h}}$ must contain the copy of *G_i _*(for otherwise, the root of the copy of *G_i _*in *T *would be a duplication, while it should be a speciation). Take any *h *such that 1 *≤ h ≤ k*. We claim that *V_h _*= {*v_i _*: $T_{x_{h}}$ contains the copy of *G_i_*} forms an inpedendent set. Since $T_{x_{h}}$ contains only speciations, it cannot contain genes from the same species. Thus for any *G_i_*, *G_j _*contained in $T_{x_{h}}$ we must have *v_i_v_j _*∉ *E*, as otherwise *G_i _*and *G_j _*would share a gene from the same species. Therefore *V_h _*is an independent set. Thus {*V*_1_,..., *V_k _*} form a *k*-coloring of *H*, and the proof is completed.   □

It is interesting to note that this does not show the NP-hardness of the special case in which the input trees are only triplets. Indeed, a tree *G_i _*created in this reduction has as many leaves as the number of neighbors of its corresponding vertex *v_i_*. Therefore, if *H *is a cubic graph (ie. 3-regular), one can generate an input with only triplets. However, deciding if a cubic graph is *k*-colorable can be done in linear time, and thus the triplets case cannot be shown NP-hard through this reduction. The 3-colorability problem is NP-hard on 4-regular graph though, showing the NP-hardness of the problem on input trees having at most 4 leaves.

## Conclusion

We introduce the supergenetree problem which aims at constructing a supertree that displays a set of input gene trees while minimizing the reconciliation cost with respect to an input species tree. This problem is a natural formulation of the question of combining a set of gene trees obtained for subsets of a gene family into a full gene tree for the whole gene family.

The supergenetree problem is an extension of the classical supertree problem on a set of input leaf-labeled trees, where the input trees are gene trees and a species tree is used in order to evaluate the reconciliation/duplication cost of a supergenetree. We show how existing exact and greedy heuristic algorithms for the supertree problem can be used to devise approaches for solving the supergenetree problem. The resulting approaches have exponential worst-time complexity as the original supertree algorithms.

We show that the supergenetree problem for the duplication cost is NP-hard to approximate within a factor essentially better than *n*, and this complexity remains the same even when the problem is restricted, in a greedy approach, to finding a supertree with a minimum number of duplications before each speciation of the species tree. We also consider a restriction of the supergenetree problem relevant to many biological applications where subsets of orthologs are studied separately and then amalgamated into a single tree. Even this restriction is shown to be NP- Complete. The reconciliation cost remains to be studied, although we conjecture all of the above mentioned problems are hard in this case also.

These negative complexity results are not surprising though as they extend an already large set of problems related to supertrees that are known to be NP-hard. We think that appropriate heuristics for various classes of input trees are worth to be considered in future projects. Removing the assumption that the input gene trees are compatible would also lead to new interesting problems. A promising avenue would be to consider constructive FPT algorithms that can be integrated in greedy heuristics or dynamic programming algorithms. Also other restrictions on the input gene trees can be explored, hopefully leading to polynomial problems. Constructing gene trees by amalgamating smaller trees for subsets of orthologous genes is a natural way of constructing large trees that would benefit from a thorough theoretical and algorithmic analysis.

## Competing interests

The authors declare that they have no competing interests.

## Authors' contributions

ML, AO, NE devised the proofs and algorithms and wrote the paper.

## Declarations

Publication of this work is funded by the Natural Sciences and Engineering Research Council of Canada (NSERC), Fonds de Recherche Nature et Technologies of Quebec (FRQNT) and the Canada Research Chair (CRC) in Biological and Computational Biology.

This article has been published as part of *BMC Bioinformatics *Volume 16 Supplement 14, 2015: Proceedings of the 13th Annual Research in Computational Molecular Biology (RECOMB) Satellite Workshop on Comparative Genomics: Bioinformatics. The full contents of the supplement are available online at http://www.biomedcentral.com/bmcbioinformatics/supplements/16/S14.
